# Application of Near-Infrared Spectroscopy Analysis Technology to Total Nucleosides Quality Control in the Fermented Cordyceps Powder Production Process

**DOI:** 10.1155/2020/8850437

**Published:** 2020-11-28

**Authors:** Tiannv Shi, Yongmei Guan, Lihua Chen, Shiyu Huang, Weifeng Zhu, Chen Jin

**Affiliations:** Key Laboratory of Modern Chinese Medicine Preparation, Ministry of Education, Jiangxi University of Traditional Chinese Medicine, Nanchang 330004, China

## Abstract

Product quality control is a prerequisite for ensuring safety, effectiveness, and stability. However, because of the different strain species and fermentation processes, there was a significant difference in quality. As a result, they should be clearly distinguished in clinical use. Among them, the fermentation process is critical to achieving consistent product quality. This study aims to introduce near-infrared spectroscopy analysis technology into the production process of fermented Cordyceps powder, including strain culture, strain passage, strain fermentation, strain filtration, strain drying, strain pulverizing, and strain mixing. First, high performance liquid chromatography (HPLC) was used to measure the total nucleosides content in the production process of 30 batches of fermented Cordyceps powder, including uracil, uridine, adenine, guanosine, adenosine, and the process stability and interbatch consistency were analyzed with traditional Chinese medicine (TCM) fingerprinting, followed by the near-infrared spectroscopy (NIRS) combined with partial least squares regression (PLSR) to establish a quantitative analysis model of total nucleosides for online process monitoring of fermented Cordyceps powder preparation products. The model parameters indicate that the established model with good robustness and high measurement precision. It further clarifies that the model can be used for online process monitoring of fermented Cordyceps powder preparation products.

## 1. Introduction

Fermented Cordyceps powder is made by liquid deep fermentation and processing. Studies have confirmed that the chemical constituents of fermented Cordyceps powder products are similar to those of *Cordyceps sinensis*, mainly including nucleosides, sterols, polysaccharides, and amino acids, which have anti-inflammatory, antioxidant, and immune regulation pharmacological effects and can be used as sustainable substitutes for *Cordyceps sinensis* [1–6]. At present, fermented Cordyceps powder preparation products are diverse on the market, including Jinshuibao Capsules, Jinshuibao Tablets, Bailing Capsules, Ningxinbao Capsules, Xinganbao Capsules, etc. Different strains and fermentation processes can result in significant differences in product quality [1]. Nucleosides are among the active ingredients, which can be used as a symbolic component of fermented Cordyceps powder, but only adenosine content determination and the identification of five nucleoside chromatographic peaks are found in the 2015 edition of Chinese pharmacopoeia. It is difficult to characterize the integrity of the quality attributes of nucleoside components in fermented Cordyceps powder preparation products, and thus the overall quality of the nucleoside components in fermented Cordyceps powder preparation products cannot be scientifically and effectively controlled. The content of each component in the fermentation process is influenced by a dynamic transfer process. How to control the effective components to reach a threshold value is still a problem. It is difficult to evaluate the quality of a product from only a part of the process, and the quality of the product should instead be traced based on multiple steps of production.

Traditional Chinese medicine (TCM) fingerprinting is a technology with the characteristics of integrity, fuzziness, and strong specificity that has been accepted by the Chinese pharmacopoeia for the quality process control of TCM [7]. Characteristic peaks and common peaks can be used to evaluate the quality of TCM quickly and accurately [8, 9]. Selecting the characteristic peaks and common peaks of the HPLC in the TCM fingerprint software and calculating the overlap rate, peak number, and peak intensity of each chromatogram. When the similarity is between 0.8 and 1.0, it indicates that all the chromatograms have good similarity [10]. It is reported that fingerprinting is commonly applied to the quality control of TCM products but less commonly applied to the process of quality control of TCM production. In addition, although fingerprinting produces a large amount of data, the results are not intuitive. Therefore, more chemometric methods should be combined for data processing [11, 12]. In the study of the nucleoside ingredients of fermented Cordyceps powder, fingerprinting was used only to monitor adenosine, guanosine, and uridine in the fermentation process, and the results showed that the contents of nucleosides fluctuated greatly in different processes, suggesting that quality control should be carried out at the beginning of the production of fermented Cordyceps powder [13].

As a process analysis technique (PAT), near-infrared spectroscopy (NIRS) has the characteristics of being fast and not damaging samples and is widely used in the online measurement of samples [14–17]. After the sample is scanned with a wavenumber range of 4000–12500 cm^−1^, various spectral pretreatment methods, standard normal transformation (SNV), multiplicative scatter correction (MSC), Savitzky-Golay (S-G) smoothing, first derivative, and the second derivative are used to reduce the interference of particle size, surface nonuniformity and color difference of powder samples with the spectrum [18, 19]. It is reported that NIRS is currently used only to monitor a certain component in the extraction process of TCM or the product, whereas fewer studies have reported that NIRS is used to monitor the production process chain [20–23]. In the NIRS studies on the nucleoside components of fermented Cordyceps powder, only adenosine was studied, which could not reflect the overall content of nucleoside components [24].

**Figure 1:**
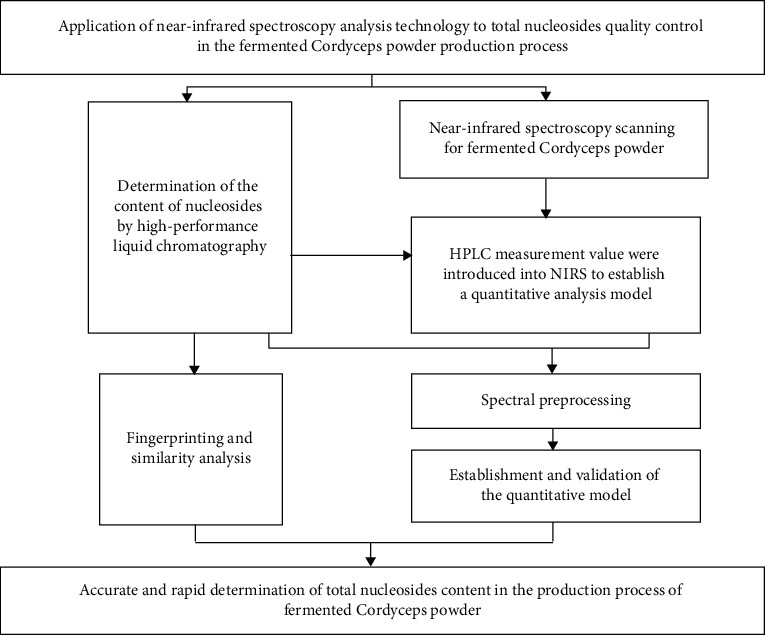
The concept of establishing a near-infrared quantitative analysis model of total nucleosides in the production process of fermented Cordyceps powder.

The trend of quality control is the transition from a single component to multiple components and from the inspection stage to overall control [17]. The quality of the preparation products is unstable, which is caused by the mutual influence of various links in the production. It is imminent to trace the source of the preparation production process. Therefore, based on TCM fingerprinting, this study approaches product quality control from a new perspective. HPLC-fingerprinting is combined with NIRS system monitoring total nucleosides of the production process of fermented Cordyceps powder, including strain culture, strain passage, strain fermentation, strain filtration, strain drying, strain pulverizing, and strain mixing, to achieve overall control of the nucleosides in the production process. The partial least squares regression (PLSR) quantitative analysis models based on HPLC and NIRS could rapidly and accurately predict the content of total nucleoside in the production process. The aim is to control the effect of the fermentation and adjust the fermentation temperature, pH, speed, etc. in the production process in real time [25], to determine an efficient and controllable fermentation process as a preliminary basis for the mass production of fermented Cordyceps powder in line with quality standards. The overall concept of this study is shown in Figure 1.

## 2. Experiment

### 2.1. Experimental Instruments and Materials

A BS124S electronic balance and a one hundred thousandth electronic analytical balance were used to weigh samples and reference materials (Sartorius, Germany). An Agilent 1260 high performance liquid chromatography (HPLC) was used to measure the content of nucleoside components (Agilent, USA). An ANTARIS II NIRS was used to acquire the spectrum of fermented Cordyceps powder (Thermo Fisher). A dual frequency computer number control ultrasonic cleaner was used to extract nucleosides (KQ-500VDE, Kunshan Ultrasonic Instrument Co., Ltd., Suzhou, China). Guanosine reference substance (batch number: 111977–201501), uridine reference substance (batch number: 20425–201508), adenosine reference substance (batch number:110879–200202), uracil reference substance (batch number: 100469–201302), and adenine reference substance (batch number: 110886–201102) were purchased from Nanchang Beta Biotechnology Co. Ltd (Nanchang, China). Fermented Cordyceps powder (30 batches were sampled in all the steps of the production process, and the total number of samples in all the steps were 210) were supplied by Jiangxi Jimin Kexin Jinshuibao Co. Ltd. (Nanchang, China).

### 2.2. Preparation of Solutions

#### 2.2.1. Preparation of Reference Solutions

Uracil, uridine, adenine, guanosine, and adenosine were accurately weighed and ultrapure water was added to a 10 mL volumetric flask to prepare reference solutions with concentrations of 366.00 *μ*g/mL, 1010.00 *μ*g/mL, 324.00 *μ*g/mL, 1217.00 *μ*g/mL, and 1474.00 *μ*g/mL, respectively.

Then, 1.5 mL of the reference solution was placed in a 10 mL volumetric flask and ultrapure water was added to the mark. The reference solution concentrations were 54.90 *μ*g/mL, 151.50 *μ*g/mL, 48.60 *μ*g/mL, 182.55 *μ*g/mL, and 221.10 *μ*g/mL.

#### 2.2.2. Preparation of Sample Solutions

Precisely 1.00 g fermented Cordyceps powder from different batches and different processes were weighed and placed in a conical flask. Then 40 mL of ultrapure water was added, and the conical flask was covered tightly, weighed, and subjected to ultrasonic processing (power 500 W, frequency 45 kHz) for 30 min. The flask was then cooled to room temperature and weighed again, and the reduced weight was replenished with ultrapure water. The conical flask was shaken until the extract was well mixed and then centrifuged (speed 4000 r/min, 10 min). The sample solution was obtained by filtering the supernatant through a 0.22-micron microporous filtration membrane [6].

### 2.3. Chromatographic Conditions

The chromatography column was an ACE Excel 5 SuperC18 (250 mm × 4.6 mm, 5 *μ*m). Solvent A was water, solvent B was methanol, and gradient elution was carried out as follows: 0–15 min, isocratic 1% B, 15–30 min, linear gradient 1%–15% B, 30–40 min, linear gradient 15%–35% B, 40–47 min, linear gradient 35%–1% B, 47–50 min, isocratic 1% B. The mobile phase flow rate was 1.00 mL/min. The detection wavelength was 260 nm, the column temperature was 30°C, and the injection volume was 10 *μ*L.

### 2.4. NIR Acquisition, Modeling, and Model Verification

#### 2.4.1. Acquisition Conditions

The spectrum was acquired by the integrating sphere mode in a region of 4000–12500 cm^−1^ with a resolution of 8 cm^−1^ and 64 scans per spectrum, temperature 25°C and humidity 40%. Repeated acquisition of 3 spectra for each fermented Cordyceps powder sample, taking the average spectrum for subsequent analysis, the spectrum results were displayed in log(1/*R*) form.

#### 2.4.2. Spectral Pretreatment

The spectrum was analyzed using TQ Analyst software. A PLSR quantitative analysis model was established to compare and analyze the MSC combined with the original spectrum, or first derivative, or second derivative and S-G smoothing spectrum results. The optimal quantitative model was selected by taking the root-mean square errors of calibration and prediction (RMSEC, RMSEP) and the correlation coefficients of calibration and prediction (*Rc*, *Rp*) as indicators. A good performance model should have high *R*, low RMSECV and RMSEP values. RMSEP was far more than RMSECV, indicating poor representability of modeling samples, while RMSEP was far less than RMSECV, indicating poor representability of validation samples [26]. When RMSEC and RMSEP are close to each other and close to 0, and the correlation coefficient is close to 1, the model is better [27].

#### 2.4.3. Division of Calibration Sets and Test Sets

The contents of the total nucleoside of different batches were arranged in descending order, ensuring that the range of calibration set contents contained the range of test set contents, and 174 calibration sets and 36 test sets were selected [28].

#### 2.4.4. Selection of Modeling Interval

According to the software recommendation for the model wavenumber range and the literature [24, 28], the wavenumber intervals were determined to be 7317–6928 cm^−1^, 6739–6476 cm^−1^, 5859–5523 cm^−1^, 4902–4818 cm^−1^, and 4740–4108 cm^−1^.

#### 2.4.5. Model Verification

The test set samples were selected as external data to verify the model, and the absolute error was used as the index evaluation model.

## 3. Results and Discussion

### 3.1. Analysis of Nucleoside Components

**Figure 2:**
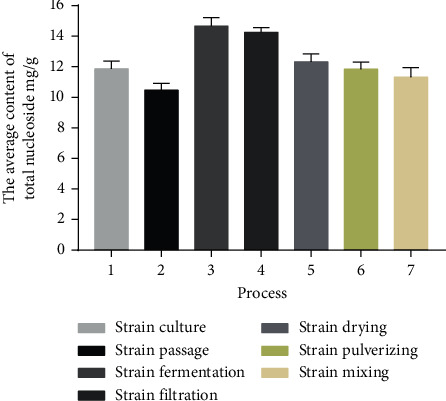
Total nucleoside average content in 30 batches of different processes.

The total nucleoside contents in 30 batches from all the steps of production processes are shown in Table 1. The contents of single nucleosides in 30 batches of different processes are shown in Table 2. The average content of total nucleosides in 30 batches of the whole process is shown in Figure 2. A one-way analysis of variance has been performed on the total nucleotide content in the process, and the results show that there is no significant difference between the strain culture and the strain mixing (*P*=0.900 > 0.05), between the strain drying and strain mixing (*P*=0.799 > 0.05), and between the strain pulverizing and strain mixing (*P*=0.647 > 0.05). Among the nucleoside components, uracil and adenine fluctuate greatly. According to the characteristics of the nucleosides content in the production process of fermented Cordyceps powder, it is suggested that the timely measurement of the nucleosides content has a certain positive effect on the quality of the final product.

### 3.2. Establishment of Fingerprints of Each Process and Similarity Analysis

**Figure 3:**
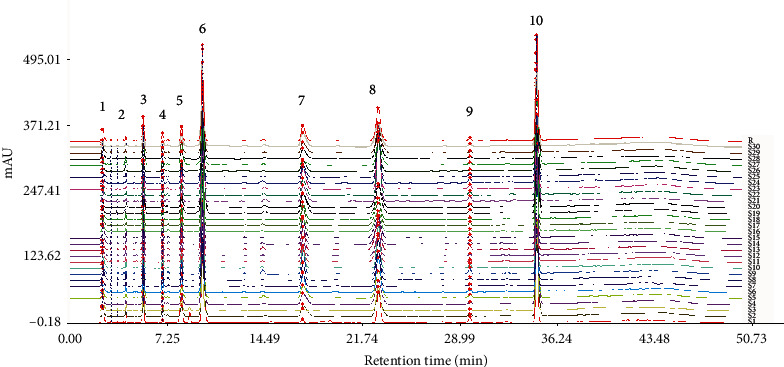
Fingerprint of 30 batches of fermented Cordyceps powder in the process of strain mixing (3. uracil, 6. uridine, 7. adenine, 8. guanosine, 10. adenosine).

The nucleoside chromatogram from all the steps of the production process detected by HPLC was introduced into the similarity evaluation system of TCM fingerprinting (2004A edition) (mean value method) recommended by the Chinese pharmacopoeia committee for similarity evaluation. The fingerprint of the production process of fermented Cordyceps powder is taken here as an example of the final fermentation process, that is, the strain mixing process, as shown in Figure 3. The production process interbatch similarity is shown in Table 3. It can be seen from Table 3 that the interbatch similarity in different processes is above 0.9 and the relative standard deviation (RSD) is less than 5%. The results show that the nucleoside components have good interbatch consistency and stability in the production process.

### 3.3. NIR Analysis Results

#### 3.3.1. Establishment of a Quantitative Model

**Figure 4:**
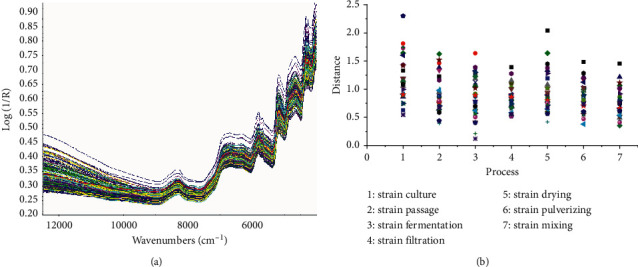
(a) The original spectrum of fermented Cordyceps powder in the production process. (b) The result of distance discrimination of 30 batches of samples in the production process.

**Figure 5:**
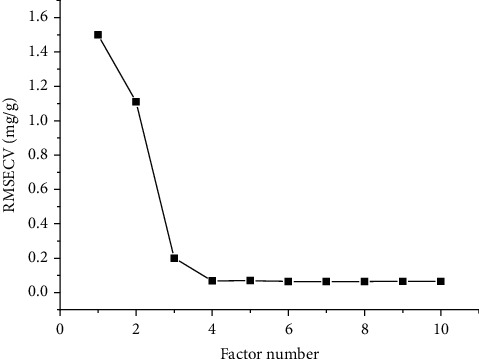
The variation of RMSECV under different principal components.

**Figure 6:**
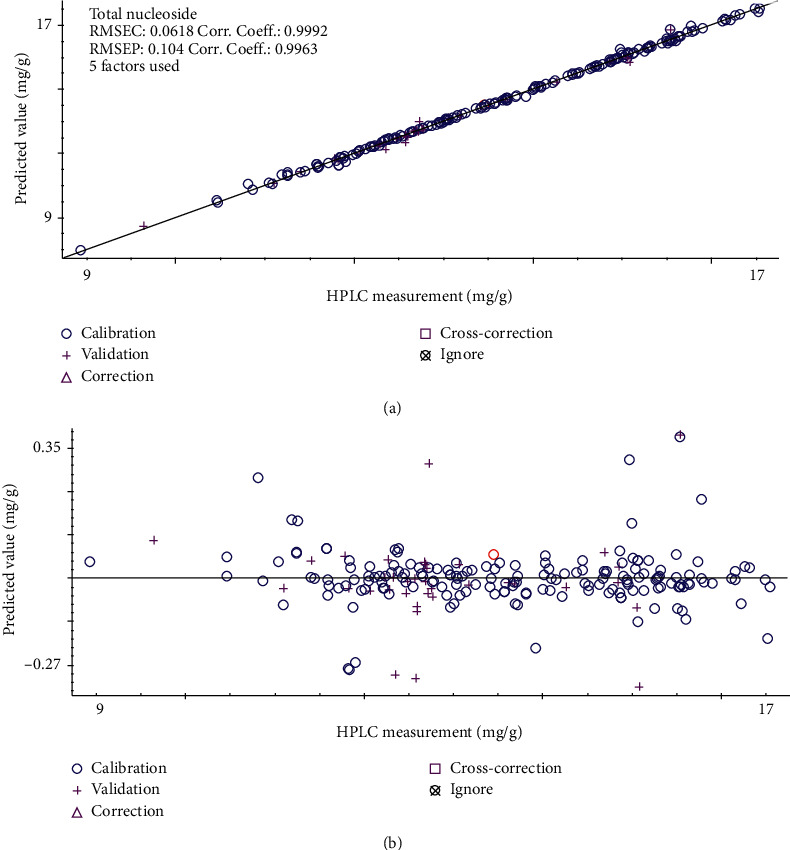
(a) PLSR quantitative analysis model of total nucleoside in the production process of fermented Cordyceps powder. (b) Absolute error scatter plot between the predicted value and the HPLC measurement of the model.

TQ Analyst analyzes the original spectrum of fermented Cordyceps powder. The original spectrum overlay diagram fermented Cordyceps powder in different processes is shown in Figure 4(a). The discriminant analysis results that takes each step as a standard, calculates the distance between the sample of each step and its own step, and judges the similarity of each step through the discriminant distance of different steps are shown in Figure 4(b). It can be seen from Figure 4(b) that the discriminant distances of different processes are within the range of 0.5–1.5, and the distribution among different batches is relatively dense, indicating that the near-infrared spectrum similarity of different processes is relatively high, and the consistency between batches is relatively good, which is consistent with the similarity analysis results of fingerprint. The pretreatment mode was MSC combined with the original spectrum, or first derivative, or second derivative and S-G smoothing spectrum results. The root-mean square error, correlation coefficients, and root-mean square errors of cross-validation (RMSECV) of the total nucleoside PLSR model under different pretreatment methods are shown in Table 4. It can be seen from Table 4 that the model parameters are not improved. The total nucleoside PLSR quantitative analysis model was established by using the original spectrum of fermented Cordyceps powder. The variation of RMSECV under different principal components is shown in Figure 5. As the number of principal components increases, RMSECV shows a decreasing trend. When the principal component is greater than 5, RMSECV is basically stable, so the number of principal components is 5. According to the model results, RMSEC = 0.0618 mg/g, RMSEP = 0.104 mg/g, *Rc* = 0.9992, *Rp* = 0.9963 and RMSECV = 0.0692 mg/g, the results show that the established model with good robustness, high measurement precision, and the model can be used to predict the total nucleosides content in fermented Cordyceps powder production process. The PLSR quantitative analysis model of total nucleosides in the production process of fermented Cordyceps powder is shown in Figure 6.

#### 3.3.2. Model Verification

The predicted values by the model and the HPLC measurement values for 36 samples are shown in Table 5. Table 5 shows that the absolute error ranges from −0.2550 to 0.6691. The Tukey test results (*P*=0.949 > 0.05) indicate that the model with high measurement precision.

## 4. Discussion

In this study, the contents of different processes and batches of nucleosides were measured by HPLC. The HPLC fingerprint was introduced into the similarity evaluation system of TCM fingerprinting (2004A edition), and the fingerprint of nucleoside ingredients in the fermented Cordyceps powder was obtained. The similarity evaluation showed that the similarity of the production process in 30 batches was above 0.9, indicating that the quality consistency of nucleoside components among different batches was good, relatively uniform, and stable. The total nucleosides content changes dynamically within the threshold range during the production process of fermented Cordyceps powder preparations. It is difficult to reflect the overall quality of the product only according to the content of a single ingredient. The results of the total nucleosides content in different processes showed a decreasing trend in the strain passage process probably because a small number of strains were selected and inoculated during the passage of the oblique culture medium, and the strain activity was low. However, the pH value of the fermentation environment was changed due to the generation of metabolites and the utilization of nitrogen sources in the later stage of fermentation during the strain fermentation, so that the total nucleosides content showed an increasing trend in this process, and basically returned to the initial content from the strain drying process.

During the establishment of the PLSR quantitative model of total nucleosides in the production process of fermented Cordyceps powder preparation products, the near-infrared spectrum overlay diagram of different processes shows that the map distribution is relatively dense, and in the qualitative process of different processes, the discriminant distance is more distributed between 0.5–1.5, indicating that the similarity of different steps is good and the production process is stable. Compared with the traditional method for determining nucleoside content [6], this study uses the characteristics of NIRS to be accurate and rapid, based on the specific operating unit in the production process of fermented Cordyceps powder preparation products, the total nucleoside PLSR quantitative analysis model for the production process that covering each operation unit was established, which is superior to the determination of the active ingredients in the single extraction process of some Chinese herbal medicines or products described in the literature [13, 20–24]. It solves the key problem that the quality of fermented Cordyceps powder preparation products cannot be controlled as a whole based on only the adenosine in the existing standard. The model parameters and model verification results showed that the model established in this study could be used to predict the total nucleosides content in the production process.

## 5. Conclusion

On the one hand, this paper uses HPLC-fingerprinting to study the quality control of total nucleosides in the production process of fermented Cordyceps powder preparation products. On the other hand, the NIRS combined with PLSR is used to establish a total nucleoside quantitative analysis model for the production process, aiming to accurately and quickly measure the total nucleosides content of the production process, solving the problems of traditional methods requiring preprocessing steps, long time, etc., and provide a reference for industrial rapid determination of total nucleoside content of fermented Cordyceps powder preparations products.

## Figures and Tables

**Table 1:** Total nucleoside content in 30 batches of different processes.

Different processes	Total nucleoside content (mg/g)	Total nucleoside average content (mg/g)
Strain culture	10.91∼12.66	11.86
Strain passage	8.99∼11.24	10.47
Strain fermentation	13.91∼16.52	14.66
Strain filtration	13.55∼14.83	14.26
Strain drying	11.19∼12.98	12.33
Strain pulverizing	10.89∼12.60	11.85
Strain mixing	10.01∼12.32	11.33

**Table 2:** Contents of single nucleosides in 30 batches of different processes.

Different processes	Uracil (mg/g)	Uridine (mg/g)	Adenine (mg/g)	Guanosine (mg/g)	Adenosine (mg/g)
Strain culture	0.04∼0.15	3.69∼5.22	0.08∼0.30	3.66∼5.05	2.16∼3.47
Strain passage	0.03∼0.17	3.23∼4.77	0.06∼0.20	3.35∼5.16	1.20∼3.06
Strain fermentation	0.02∼0.19	4.34∼5.78	0.30∼0.92	5.50∼7.03	2.00∼3.02
Strain filtration	0.08∼0.19	4.55∼5.37	0.28∼0.59	5.52∼6.51	2.23∼3.19
Strain drying	0.08∼0.19	3.33∼5.67	0.12∼0.44	4.42∼5.70	1.22∼3.05
Strain pulverizing	0.09∼0.19	3.36∼5.34	0.20∼0.65	4.09∼5.36	1.72∼3.21
Strain mixing	0.10∼0.23	3.24∼4.60	0.25∼0.53	3.72∼4.96	1.89∼3.33

**Table 3:** Batch similarity of fermented Cordyceps powder in the production process.

Process	Maximum similarity	Minimum similarity	RSD (%)
Strain culture	0.999	0.908	4.04
Strain passage	0.997	0.973	1.36
Strain fermentation	0.999	0.910	2.26
Strain filtration	0.999	0.910	1.63
Strain drying	0.999	0.900	2.25
Strain pulverizing	0.999	0.953	1.16
Strain mixing	0.999	0.922	1.52

**Table 4:** Root-mean square error and correlation coefficient of different pretreatment methods of total nucleosides in fermented Cordyceps powder.

Optical path type	Spectral pretreatment	RMSEC (mg/g)	*R*_*c*_	RMSEP (mg/g)	*R*_*p*_	RMSECV (mg/g)	Principal components
MSC	Original spectrum	0.0618	0.9992	0.1040	0.9963	0.0692	5
	First derivative	0.1090	0.9976	0.1640	0.9912	0.1630	9
	Second derivative	0.2130	0.9906	0.7840	0.8106	0.8400	9
	S-G	0.0618	0.9992	0.1040	0.9963	0.0692	5

**Table 5:** Comparison between the HPLC measurement and the predicted value of the total nucleosides content in fermented Cordyceps powder.

Sample number	HPLC measurement value (mg/g)	Predicted value (mg/g)	Absolute error	Sample number	HPLC measurement value (mg/g)	Predicted value (mg/g)	Absolute error
1	13.6003	13.6343	0.0340	19	12.5265	12.2798	−0.2467
2	10.7855	10.7735	−0.0120	20	15.8988	15.8882	−0.0106
3	12.3524	12.5474	0.1950	21	14.0509	13.9831	−0.0678
4	9.6604	9.6078	−0.0526	22	13.2420	13.2430	0.0010
5	12.5007	12.4406	−0.0601	23	14.9328	14.9207	−0.0121
6	14.2815	14.1950	−0.0865	24	12.2460	12.2931	0.0471
7	13.3516	13.3974	0.0458	25	13.3462	13.3473	0.0011
8	12.3292	12.3686	0.0394	26	14.5907	14.6488	0.0581
9	12.5759	12.3209	−0.2550	27	12.6324	12.4301	−0.2023
10	14.1574	14.0550	−0.1024	28	10.8168	11.4859	0.6691
11	14.5175	14.4686	−0.0489	29	12.8656	12.8305	−0.0351
12	13.2388	13.5542	0.3154	30	15.0292	15.0062	−0.0230
13	13.2149	13.1960	−0.0189	31	14.1709	14.2000	0.0291
14	13.0992	13.1475	0.0483	32	14.4696	14.5269	0.0573
15	12.7264	12.9103	0.1839	33	13.3943	13.5578	0.1635
16	10.3291	10.3320	0.0029	34	11.4855	11.4175	−0.0681
17	12.0484	12.1714	0.1230	35	10.7893	10.8572	0.0679
18	12.5426	12.4723	−0.0703	36	13.3516	13.3974	0.0458

There is no significant difference between the HPLC measurement values and the predicted values (*P*=0.949 > 0.05).

## Data Availability

The data used to support the findings of this study are included within the article.

## References

[1] Wu Y., Chen L. H., Zhu W. F. (2017). Research progress on quality standards of fermented Cordyceps powder products. *Chinese Journal of Experimental Traditional Medical Formulae*.

[2] Liu Y., Wang J., Wang W., Zhang H., Zhang X., Han C. (2015). The chemical constituents and pharmacological actions of *Cordyceps sinensis*. *Evidence-Based Complementary and Alternative Medicine*.

[3] Zhang H., Li Y., Mi J. (2017). GC-MS profiling of volatile components in different fermentation products of *Cordyceps sinensis* mycelia. *Molecules*.

[4] Nakamura K., Shinozuka K., Yoshikawa N. (2015). Anticancer and antimetastatic effects of cordycepin, an active component of Cordyceps sinensis. *Journal of Pharmacological Sciences*.

[5] Chen L. H., Yang M. J., Guan Y. M., Zhu W.-F., Huang H.-L. (2014). Identification of nucleosides and nucleobases from cultured *Cordyceps militaris*. *Natural Product Communications*.

[6] Chen L. H., Wu Y., Guan Y. M., Jin C., Zhu W.-F., Yang M. (2018). Analysis of the high-performance liquid chromatography fingerprints and quantitative analysis of multicomponents by single marker of products of fermented *Cordyceps sinensis*. *Journal of Analytical Methods in Chemistry*.

[7] Zhang H., Chen Y., Wang J. N., Jiang H.-J., Shen X.-W., Yan J.-Z. (2018). Application of fingerprint technology in quality evaluation and process control of traditional Chinese medicine formula granules. *Chinese Journal of Traditional Materia Medica*.

[8] Cai Y., Li X., Li M. (2015). Traceability and quality control in traditional Chinese medicine: from chemical fingerprint to two-dimensional barcode. *Evidence-Based Complementary and Alternative Medicine*.

[9] Liu C., Guo D.-A., Liu L. (2018). Quality transitivity and traceability system of herbal medicine products based on quality markers. *Phytomedicine*.

[10] Guan H. Y., Li L., Liu X. (2011). Study on similarity algorithm of traditional Chinese medicine fingerprints. *Chinese Journal of Experimental Traditional Medical Formulae*.

[11] Zhang Y., Yang F., Zhang J. (2019). Quantitative fingerprint and quality control analysis of compound liquorice tablet combined with antioxidant activities and chemometrics methods. *Phytomedicine*.

[12] Yin F., Li L., Li X. (2016). Decoction process optimization and quality evaluation of Yi-Huang decoction by HPLC fingerprint analysis. *Acta Poloniae Pharmaceutica*.

[13] Zhang X. T., Zhong C. Z., Yang M. (2020). Establishment of quality control method in production of Jinshuibao capsules. *Chinese Journal of Experimental Traditional Medical Formulae*.

[14] Boiret M., Chauchard F. (2017). Use of near-infrared spectroscopy and multipoint measurements for quality control of pharmaceutical drug products. *Analytical and Bioanalytical Chemistry*.

[15] Suo T., Wang H., Shi X. (2018). Combining near infrared spectroscopy with predictive model and expertise to monitor herb extraction processes. *Journal of Pharmaceutical and Biomedical Analysis*.

[16] Liu R., Li L., Yin W. (2017). Near-infrared spectroscopy monitoring and control of the fluidized bed granulation and coating process-a review. *International Journal of Pharmaceutics*.

[17] Zhang N., Xu B., Chen Y. B. (2018). The quality of traditional Chinese medicine originates from the design method and application:the whole process quality control. *World Chinese Medicine*.

[18] Liu R., Sun Q., Hu T. (2018). Multi-parameters monitoring during traditional Chinese medicine concentration process with near infrared spectroscopy and chemometrics. *Spectrochimica Acta Part A: Molecular and Biomolecular Spectroscopy*.

[19] Alarm M. A., Shi Z., Drennen J. K., Anderson C. A. (2017). In-line monitoring and optimization of power flow in a simulated continuous process using transmission near infrared spectroscopy. *International Journal of Pharmaceutics*.

[20] Skou P. B., Berg T. A., Aunsbjerg S. D., Thaysen D., Rasmussen M. A., Van Den Berg F. (2017). Monitoring process water quality using near infrared spectroscopy and partial least squares regression with prediction uncertainty estimation. *Applied Spectroscopy*.

[21] Yang Y., Wang L., Wu Y. (2017). On-line monitoring of extraction process of Flos Lonicerae Japonicae using near infrared spectroscopy combined with synergy interval PLS and genetic algorithm. *Spectrochimica Acta Part A: Molecular and Biomolecular Spectroscopy*.

[22] Ma Y., He H., Wu J. (2018). Assessment of polysaccharides from mycelia of genus ganoderma by mid-infrared and near-infrared spectroscopy. *Scientific Reports*.

[23] Wang H., Suo T., Wu X. (2018). Near infrared spectroscopy based monitoring of extraction processes of raw material with the help of dynamic predictive modeling. *Spectrochimica Acta Part A: Molecular and Biomolecular Spectroscopy*.

[24] Xu N., Wei W., Ren B. (2012). Near-infrared spectroscopy quantitative analysis and band selection of aqueous adenosine from fermented *Cordyceps sinensis* powder. *Spectroscopy and Spectral Analysis*.

[25] Wang L.-Y., Cheong K.-L., Wu D.-T., Meng L.-Z., Zhao J., Li S.-P. (2015). Fermentation optimization for the production of bioactive polysaccharides from *Cordyceps sinensis* fungus UM01. *International Journal of Biological Macromolecules*.

[26] Yang L. X., Zhang Y. X., Feng W. H. (2015). Establishment of simultaneous quantitative model of five alkaloids from Corydalis Rhizoma by near-infrared spectrometry. *China Journal of Chinese Materia Medica*.

[27] Wang H., Tian H. Y., Zhang S. L. (2017). Portable medium-wave near-infrared spectrometer online non-destructive testing of fresh pork cholesterol. *Spectroscopy and Spectral Analysis*.

[28] Wang S. P., Liu Q. P., Liu H. M. (2013). Finding the authenticity of Jinshuibao capsule by near-infrared spectroscopy. *Chinese Pharmaceutical Affairs*.

